# Highly efficient synergistic activity of an α-L-arabinofuranosidase for degradation of arabinoxylan in barley/wheat

**DOI:** 10.3389/fmicb.2023.1230738

**Published:** 2023-11-03

**Authors:** Jiaqi Wen, Ting Miao, Abdul Basit, Qunhong Li, Shenglin Tan, Shuqing Chen, Nuraliya Ablimit, Hui Wang, Yan Wang, Fengzhen Zheng, Wei Jiang

**Affiliations:** ^1^State Key Laboratory of Animal Biotech Breeding, College of Biological Sciences, China Agricultural University, Beijing, China; ^2^Department of Microbiology, University of Jhang, Jhang, Punjab, Pakistan; ^3^Little Tiger Biotechnology Company Limited, Hangzhou, Zhejiang, China; ^4^College of Biological and Environmental Engineering, Zhejiang Shuren University, Hangzhou, China

**Keywords:** α-L-arabinofuranosidase, enzyme synergism, arabinoxylan, site-directed mutagenesis, catalytic residues

## Abstract

Here, an α-L-arabinofuranosidase (termed TtAbf62) from *Thermothelomyces thermophilus* is described, which efficiently removes arabinofuranosyl side chains and facilitates arabinoxylan digestion. The specific activity of TtAbf62 (179.07 U/mg) toward wheat arabinoxylan was the highest among all characterized glycoside hydrolase family 62 enzymes. TtAbf62 in combination with endoxylanase and β-xylosidase strongly promoted hydrolysis of barley and wheat. The release of reducing sugars was significantly higher for the three-enzyme combination relative to the sum of single-enzyme treatments: 85.71% for barley hydrolysis and 33.33% for wheat hydrolysis. HPLC analysis showed that TtAbf62 acted selectively on monosubstituted (C-2 or C-3) xylopyranosyl residues rather than double-substituted residues. Site-directed mutagenesis and interactional analyses of enzyme–substrate binding structures revealed the catalytic sites of TtAbf62 formed different polysaccharide-catalytic binding modes with arabinoxylo-oligosaccharides. Our findings demonstrate a “multienzyme cocktail” formed by TtAbf62 with other hydrolases strongly improves the efficiency of hemicellulose conversion and increases biomass hydrolysis through synergistic interaction.

## 1. Introduction

Lignocellulosic biomass is an important potential sustainable source for a wide range of biofuels, chemicals, and organic materials produced by second-generation biorefineries (Premalatha et al., 2015; Sudarsanam et al., 2018; Basit et al., 2018b). Hemicellulose, the second most abundant polysaccharide (after cellulose), is a key component of plant biomass and is heavily utilized in biomass-based sustainability frameworks (Schädel et al., 2010; Scheller and Ulvskov, 2010; Ubando et al., 2020). Arabinoxylan is the main component of hemicellulosic polysaccharides, which are widely present in cereal brans and grains (De Backer et al., 2010; Lin et al., 2021). The arabinoxylan polysaccharide backbone is polymerized by β-1,4-xylopyranosyl residues, and the side chains are substituted by arabinofuranosyl residues through α-1,3 and/or α-1,2 linkages (Oliveira et al., 2020; Đorđević et al., 2021). Degradation of arabinoxylan involves synergistic action of multiple enzymes, in addition to xylanases and xylosidases that act on the xylan backbone (Gil-Durán et al., 2018; Saldarriaga-Hernández et al., 2020). α-L-arabinofuranosidases (ABFs) are auxiliary enzymes that participate in debranching of arabinofuranosyl substitutions from xylopyranosyl residues, thereby enhancing accessibility of other hemicellulases and degradation efficiency.

ABFs (also known as arabinoxylan-arabinofuranohydrolases; AXHs) hydrolyze carbohydrates containing arabinofuranosyl residues and are categorized into six glycoside hydrolase (GH) families in the carbohydrate-active enzymes (CAZy) database: GH2, GH3, GH43, GH51, GH54, GH62, and GH159 (Yan et al., 2021; Baudrexl et al., 2022). Family GH62 contains mainly ABFs found exclusively in fungi and bacteria (Siguier et al., 2014). ABFs catalyze the hydrolysis of arabinose from the non-reducing ends and are also categorized into three types based on their mode of action on differing substrates. AXHs-m act on monosubstituted (C-2 or C-3) xylopyranosyl residues, AXHs-d3 release C-3-linked L-arabinofuranosyl in double substitutions, and AXHs-md display dual activity on mono-substituted and double-substituted xylopyranosyl residues (Poria et al., 2020).

The efficient degradation of arabinoxylan is of great interest because it facilitates the production of biofuels and probiotics and many other important processes in the chemical and food industries (Gírio et al., 2010; Cheng et al., 2019). Incomplete degradation of arabinoxylan has various undesirable consequences, including reduced filtration rate, excessive consumption of time and resources, and substandard quality of end products (Li et al., 2020; Kaushal et al., 2021; Yu et al., 2021). Enzymatic digestion of arabinoxylan is often made less efficient by arabinofuranosyl substitutions on the xylan backbone, which inhibit catalysis by xylanases. Different types of hydrolytic ABFs should be available for efficient degradation, according to whether arabinofuranosyl residues are attached to single- or double-substituted xylose residues (McKee et al., 2012). Improvement of ABFs as accessory enzymes in biomass conversion processes is a high research priority, and the development of novel ABFs with strong enzymatic properties and arabinoxylan degradation capacity is important for many industrial processes.

Here, we described cloning and characterization of a novel GH62 ABF (termed TtAbf62) from the thermophilic fungus *Thermothelomyces thermophilus* in the methylotrophic yeast *Pichia pastoris* (Looser et al., 2015). *Thermothelomyces thermophilus* is an excellent source of hydrolytic enzymes for biomass degradation (Higasi et al., 2021). We enhanced the enzyme activity of TtAbf62 in *P. pastoris* by signal peptide modification engineering and high-density fermentation. TtAbf62 displayed its maximal activity toward natural substrate arabinoxylan and efficient synergism with endoxylanase (Taxy11; Zheng et al., 2020) and β-xylosidase (Ttxy43; Basit et al., 2019) in biomass conversion. Homology modeling and interactional analyses of enzyme–substrate binding structures of TtAbf62 clarified the catalytic mechanism of its AXH-m2,3-specific activity pattern toward arabinoxylo-oligosaccharides. Our findings provide a practical basis for designing “cocktails” of degrading enzymes for more efficient biomass conversion.

## 2. Materials and methods

### 2.1. Strains, reagents, and chemicals

*Escherichia coli* strain DH5α (used as a host for recombinant plasmids, pPICZαA) and *P. pastoris* strain X-33 (used as a host for gene expression) are maintained in our laboratory. PCR reagents and DNA markers were purchased from Takara (Dalian, China). Plasmid extraction kits were purchased from Tiangen (Beijing). Restriction endonucleases were purchased from New England Biolabs (Beverly, MA, United States; “NEB”). Protein markers were purchased from SMOBIO Technology (Hsinchu, Taiwan). Substrates such as wheat arabinoxylan (WAX; low-viscosity; arabinose/xylose rates ranging from 0.19 to 0.31), rye arabinoxylan (RAX; high-viscosity), beechwood xylan (BWX), 3^2^-α-L-arabinofuranosyl-xylobiose (A^3^X), 2^3^-α-L-arabinofuranosyl-xylotriose (A^2^XX), 2^3^,3^3^-di-α-L-arabinofuranosyl-xylotriose (A^2 + 3^XX), and 3^3^-α-L-arabinofuranosyl-xylotetraose (XA^3^XX) were purchased from Megazyme (Wicklow, Ireland). Sodium carboxymethyl cellulose (CMC), locust bean gum (LBG; from *Ceratonia siliqua* seeds), 4-nitrophenyl α-L-arabinofuranoside (*p*NPAf), 4-nitrophenyl β-D-xylopyranoside (*p*NPX), 4-nitrophenyl β-D-glucopyranoside (*p*NPG), and 4-nitrophenyl β-D-cellobioside (*p*NPC) were purchased from Sigma-Aldrich (St. Louis, MO, United States). Konjac glucomannan (KGM) was purchased from Youpintang Biotech Co. (Beijing). Pure xylobiose, xylotriose, xylotetraose, and L-arabinose were purchased from Yuanye Biotechnology Co. (Shanghai). All other chemicals used were of analytical grade and commercially available.

### 2.2. Construction of recombinant plasmids and expression of TtAbf62

**Figure 1:**
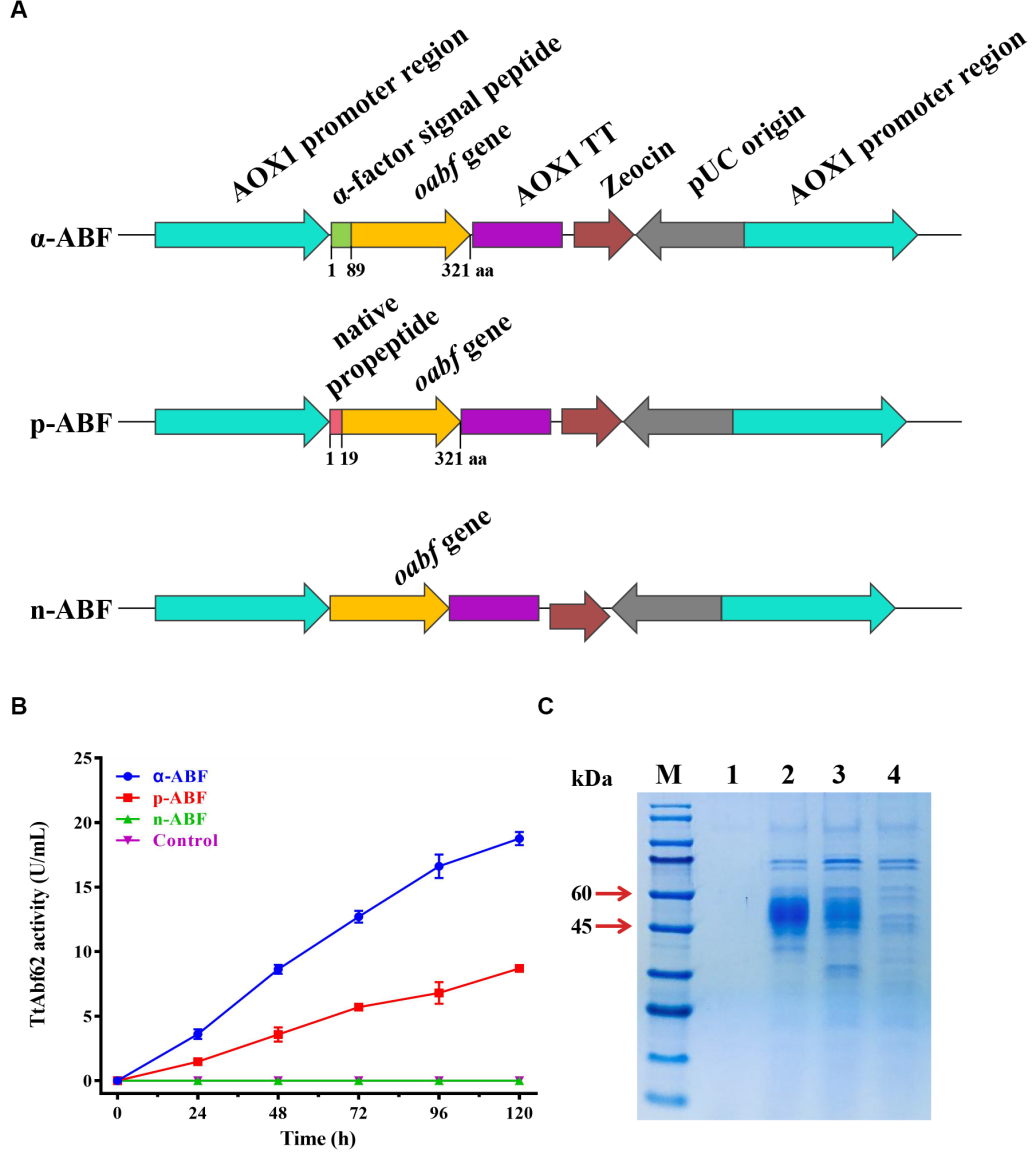
Signal peptide modification engineering of TtAbf62 constructs. **(A)** TtAbf62 constructs with differing signal peptides (schematic): α-ABF (optimized *abf* gene with α-factor signal peptide), p-ABF (optimized *abf* gene with native propeptide), n-ABF (optimized *abf* gene without signal peptide). **(B)** Comparison of α-ABF, p-ABF, n-ABF, and control (X-33) strains in terms of TtAbf62 activity levels toward wheat arabinoxylan. Assays were performed in triplicate. **(C)** SDS-PAGE gel images of each construct. M, molecular marker; 1, control (X-33); 2, α-ABF; 3, p-ABF; 4, n-ABF.

*poabf* (codon-optimized gene containing native propeptide; 966 bp) and *oabf* (not containing native propeptide) from *T. thermophilus* (ATCC 42464) were PCR-amplified using specific primer pairs (Supplementary Table 1). After sequencing validation, products were purified, digested with *Eco*RI and *Kpn*I, ligated into vectors pPICZαA/pPICZ A (without α-factor signal peptide), and pretreated with corresponding enzymes. Recombinant plasmids containing *oabf* and *poabf* genes (termed pPICZα-*oabf*, pPICZp-*oabf*, and pPICZ-*oabf*) were transformed into *E. coli* (DH5*α*), and positive transformants were screened by culturing in Zeocin (Invitrogen; Carlsbad, CA, United States) LB plates. Recombinant plasmids were linearized with *Sac*I, and transformed into *P. pastoris* X-33 by electroporation as per *Pichia* expression manual, to obtain engineered strains α-ABF, p-ABF, and n-ABF (Figure 1A). Vector-only control strain was generated by transforming *P. pastoris* with empty vector pPICZαA. Agar plates coated with yeast extract peptone dextrose with sorbitol (YPDS) containing 100 μg/mL Zeocin were used for the selection of transformants.

Colonies were confirmed by PCR using specific primers (Supplementary Table 1) and cultured in buffered minimal methanol medium (BMMY), as described previously (Basit et al., 2018a). Supernatant samples were analyzed using Tricine-SDS-PAGE, and protein levels were measured by the Bradford method (Bradford, 1976).

### 2.3. Enzyme activity assay

TtAbf62 enzyme activity was quantified on substrates, such as WAX, RAX, BWX, and CMC, using the 3,5-dinitrosalicylic acid (DNS) method (Miller, 1959) to measure released reducing sugars. The standard reaction was performed for 10 min at 60°C in 50 mM sodium acetate buffer (pH 5.0) containing 100 μL 1.0% (w/v) substrates (0.6% [w/v] for LBG and KGM) and 100 μL of appropriate diluted enzyme. The reaction was terminated by adding 50 μL of 1 M NaOH and 150 μL DNS, and the product was boiled for 5 min and cooled in an ice water bath. A similar reaction system with the inactivated enzyme was used as a control. The amount of reducing sugars was determined based on the absorbance at wavelength 540 nm and standard curve. One unit of enzyme activity was defined as the amount of enzyme that released 1 μmol reducing sugars per minute (Anonymous, 1979).

Hydrolysis of *p*NPG, *p*NPC, *p*NPAf, and *p*NPX was assayed by measuring the amount of p-nitrophenol (*p*NP) at the absorbance of 410 nm. The standard reaction was performed by co-incubating substrate (5 mM) and appropriate amount of diluted enzyme in 50 mM sodium phosphate buffer (pH 6.0) for 10 min at 50°C and terminated by adding 100 μL Na_2_CO_3_ (1 M). One unit enzyme activity was defined as the amount of enzyme that released 1 μmol *p*NP per minute under defined conditions (Anonymous, 1979). All assays were performed in triplicate.

### 2.4. Biochemical properties of TtAbf62

Enzymatic properties were evaluated using substrate WAX. Optimal pH, optimal temperature, thermostability, and pH stability were determined as described previously (Basit et al., 2019). Residual enzyme activity was determined for each of the above parameters.

To evaluate the effects of metal ions and various chemical reagents on enzyme activities, relative activities were determined for 12 metal ions (Ni^2+^, Co^2+^, Al^3+^, Na^+^, Mn^2+^, Zn^2+^, Cu^2+^, Ca^2+^, Fe^2+^, Mg^2+^, K^+^, and Fe^3+^; each 5 mM), 1 mM EDTA, 0.1% (w/v) SDS, and 0.05% (v/v) Tween-20 under standard assay conditions. Mixtures without addition were used as controls.

The specificity of TtAbf62 was evaluated using WAX, RAX, BWX, CMC, LBG, KGM, and *p*NP derivatives (*p*NPAf, *p*NPC, *p*NPG, and *p*NPX) as substrates, by determining relative activities as described above. For each substrate, specific activity was defined as units per mg protein.

Kinetic parameters of TtAbf62 were evaluated by measuring residual enzyme activity of the recombinant protein in the supernatant at WAX concentrations ranging from 1 to 20 mg/mL. Michaelis–Menten kinetic equation was used to calculate V_max_ (maximal catalytic velocity of enzyme) and K_m_ (substrate concentration at which enzyme achieves 50% of V_max_) values. All determinations described above were performed in triplicate.

### 2.5. High-cell-density fermentation

For fed-batch culture, a product from overnight culture (10% v/v) was inoculated into 5 L basal salt medium (BSM) and placed in a 7.5 L fermentor (Shanghai Boxing Bio-engineering Equipment Co.), with temperature maintained at 30°C and pH maintained at 5.5 by controlled supplementation of ammonium hydroxide (50% v/v). Glycerol was used initially as the sole carbon source in the medium. When the dissolved oxygen level showed a rapid increase, 50% (v/v) glycerol (12 mL PTM1 trace salts solution per liter glycerol) was added (18 mL/h/L; fed-batch phase). When glycerol was completely depleted, methanol containing 1.2% PTM1 was pumped into the fermentor by autocontrol system (methanol feeding phase). Samples of supernatant were taken at 12-h intervals for measurement of enzyme activity and OD_600_.

### 2.6. TtAbf62 deglycosylation analysis

Glycosylation sites in the sequence were predicted by the NetNGlyc 1.0 server program.^1^ Endoglycosidase H (Endo H; NEB) was used for the deglycosylation assay of the recombinant enzyme. The typical reaction procedure is as follows: 20 μg TtAbf62 mixed with denaturation buffer, heated for 10 min at 100°C, divided into two samples, treated with Endo H or not (control), incubated for 1 h at 37°C, and analyzed all reaction mixtures by SDS-PAGE.

### 2.7. TtAbf62 function in barley/wheat degradation

Hulled barley and wheat grain were crushed, digested enzymatically with α-amylase to remove starch, and subjected to starch saccharification by mixing 20% (w/v) barley/wheat with 120 U/g α-amylase and 0.4% (w/w) calcium chloride in sodium phosphate buffer (pH 6.0) at 60°C. The saccharification process was completed in 3 h. The product was run through several rinse/centrifugation cycles with deionized water until the concentration of reducing sugars was undetectable by the DNS method and stored at −20°C.

As pretreated above, degradation of barley/wheat (10% w/v) was performed as “optimum compromise allocation” by adding appropriate amount of 50 mM sodium acetate buffer (pH 6.0) in 50 mL flask, adding a combination of Taxy11 (dosage 50 U per g biomass), Ttxy43 (dosage 50 U per g biomass), and TtAbf62 (dosage 50 U per g biomass), and incubated (temperature 45°C) for 5 h under shaking condition (160 rpm). Hydrolysis products were taken at 1-h interval and centrifuged (13,800 x*g*, 4 min), and the concentration of reducing sugars was estimated by the DNS method (Miller, 1959). All enzymatic degradation reactions were performed in triplicate.

### 2.8. Hydrolytic activity of TtAbf62

Hydrolytic degradation by TtAbf62 (α-ABF) and its mutants was evaluated based on analysis of hydrolysis product components of WAX and arabinoxylo-oligosaccharides (AXOS), which are, respectively, mono- and di-substituted by arabinofuranosyl at C(O)-2 and/or C(O)-3 position (A^3^X, A^2^XX, XA^3^XX, and A^2 + 3^XX) on the xylose backbone. Hydrolysis reaction mixture (1% WAX or 5 mg/mL AXOS with a final enzyme concentration of 7.7 U/mL in 50 mM sodium acetate buffer, pH 5.0) was incubated for 1 h at 60°C (control: inactivated enzyme incubated with substrate). Standard reference components were xylobiose, xylotriose, and xylotetraose. Hydrolysis products were analyzed by HPLC: LC-20A system (Shimadzu; Kyoto) comprising RID-10A refractive index detector, CBM-20A controller, and ROA-Organic acid H^+^ (8%) column (Phenomenex; Torrance, CA, United States); mobile phase 5 mM H_2_SO_4_ (flow rate 0.6 mL/min); temperature maintained at 50°C by column heater.

### 2.9. Construction of mutants

The mature protein sequence of TtAbf62 (without the signal peptide 1–19 aa) was used to construct a 3D structure model using the automatic homology-modeling server SWISS-MODEL^2^ and AlphaFold (2.2.0); the structures were visualized with PyMOL Molecular Graphics System.^3^ The conformations of each ligand were generated using ChemSpider.^4^ The distances between each catalytic site, the side chain, and the backbone length of AXOS (A^3^X, A^2^XX, XA^3^XX, and A^2 + 3^XX) were analyzed using the PyMOL program. The AXOS side chain length is shorter than the distances between corresponding catalytic sites, and these sufficient distances between the catalytic sites permit the substrates to enter the catalytic region, which is essential for the efficient degradation of the side chains. Following this principle, we simulated the optimal binding conformation using PyMOL.

TtAbf62 catalytic sites were predicted based on the SthAraf62A template structure (sequence similarity 75.75%; Protein Data Bank accession number 4O8N; Wang et al., 2014). Residues such as Glu^88^, Glu^190^, Glu^208^, Glu^258^, Asp^31^, Asp^38^, Asp^49^, Asp^63^, Asp^111^, Asp^138^, Asp^155^, Asp^253^, Asp^265^, and Asp^289^ were identified as potential active catalytic sites based on multiple sequence alignment with SthAraf62A (Supplementary Figure 1). The multiple-sequence alignment was produced using Clustal Omega,^5^ and the results were visualized with Jalview software.^6^ TtAbf62 mutant strains were generated by site-directed mutagenesis approach, and strains corresponding to active catalytic residues were designated as E88A, E190A, E208A, E258A, D31A, D38A, D49A, D63A, D111A, D138A, D155A, D253A, D265A, and D289A. Mutant genes were amplified using plasmid pPICZα-*oabf* and corresponding primer pairs (Supplementary Table 1). Recombinant plasmids were constructed, sequenced, validated, and expressed, and enzyme activities were assayed as described above. The initial vector pPICZα-*oabf* was transformed into *P. pastoris* as control. Enzyme activities were evaluated using substrate WAX as described above.

## 3. Results

### 3.1. Sequence analysis and expression of TtAbf62 in *Pichia pastoris*

Sequence analysis based on the NCBI^7^ and CAZy^8^ databases revealed a gene sequence (designated as *poabf*; 966 bp, Gene ID: 11507086) from *T. thermophilus* (ATCC 42464) encoding an ABF (TtAbf62) categorized in the family GH62. TtAbf62 had a theoretical molecular mass of 35 kDa and predicted pI 5.18, according to an analysis by the ProtParam tool of the ExPASY program.^9^ *poabf* gene encodes a protein of 321 amino acids including a predicted N-terminal signal peptide of 1 to 19 amino acids (MRPTRPGIVLLATATSVAG) as it is predicted by SignalP 5.0 server program.^10^ The enzyme belonged to the family GH62 according to the CAZy database classification. The deduced amino acid sequence of TtAbf62 showed high identity (76.74 and 70.86%) with two GH62 ABFs from *Thermothielavioides terrestris* NRRL 8126 (XM_003650950.1, XP_003657290.1; Berka et al., 2011), followed by identities of 65.02 and 62.89% with GH62 ABFs from *Aspergillus aculeatus* (ARA; KT003533.1; Laothanachareon et al., 2015) and *Scytalidium thermophilum* strain CBS 625.91 (StAbf62A; KJ545572; Kaur et al., 2015), respectively.

*Pichia pastoris* is generally considered the most effective industrial host strain for the heterologous production of recombinant proteins. To enhance the efficiency of heterologous protein expression, a strategy of signal peptide modification was applied. Recombinant strains were constructed based on the native engineered strain (p-ABF) by deletion of native propeptide (termed as n-ABF) or its replacement using α-factor signal peptide (termed as α-ABF), and the effect of signal peptide replacement on the TtAbf62 activity was evaluated (Figures 1A,B). Following transformation and PCR verification (Supplementary Figure 2A) of linearized design of expression vectors pPICZα-*oabf*, pPICZp-*oabf*, and pPICZ-*oabf*, respective recombinant strains α-ABF, p-ABF, and n-ABF were obtained.

Enzyme activities of ABF during a 120-h period were compared for two substrates. Extracellular enzyme activity on substrate WAX for α-ABF (18.8 U/mL) was 2.2-fold higher than that for p-ABF (8.7 U/m); n-ABF displayed no enzyme activity (Figure 1B). The results with substrate 4-nitrophenyl α-L-arabinofuranoside (*p*NPAf) were similar to those with WAX (Supplementary Figure 2B), and protein secretion was higher for α-ABF (Figure 1C). Target proteins in α-ABF and p-ABF migrated with similar apparent molecular weights (45–60 kDa; Figure 1C). These values were higher than the predicted molecular weight (35 kDa), presumably as a result of glycosylation.

### 3.2. High-density fermentation and deglycosylation analysis

**Figure 2:**
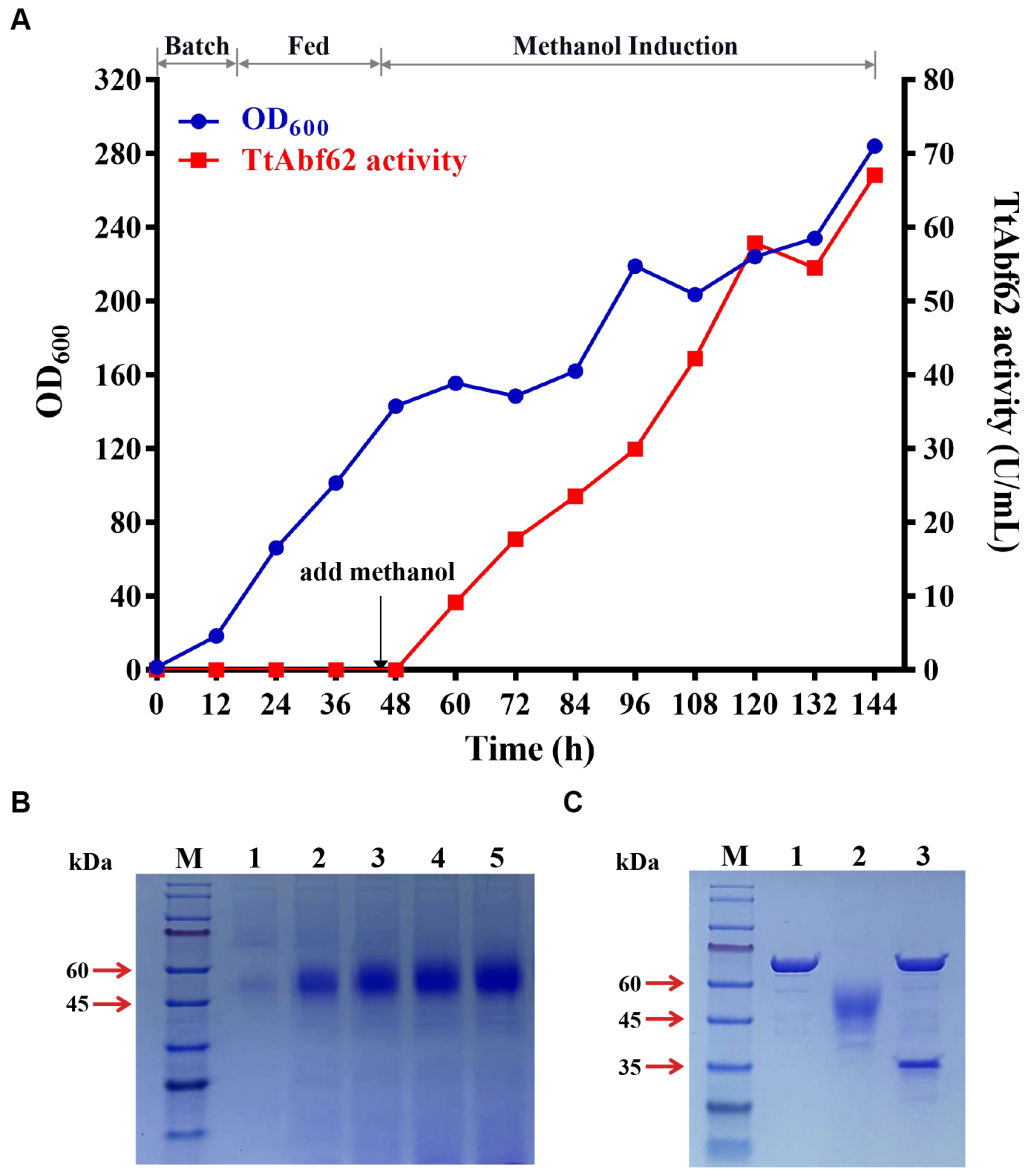
Fed-batch fermentation of engineered strain α-ABF in 7.5-L fermentor, and protein deglycosylation analysis. **(A)** Cell density (OD_600_) and extracellular TtAbf62 activity of α-ABF in fermentor. **(B)** SDS-PAGE analysis at various induction time points. M, molecular marker; 1, 36 h; 2, 72 h; 3, 96 h; 4, 120 h; 5, 144 h. **(C)** Deglycosylation analysis of TtAbf62 on SDS-PAGE. M, molecular marker; 1, Endo H; 2, TtAbf62 expressed by α-ABF; 3, deglycosylated TtAbf62 expressed by α-ABF.

Large-scale fermentation of engineered strain α-ABF was performed in a 7.5-L fermentor. Accumulation of cellular biomass resulted in 46-h culturing and feeding with glycerol as the sole carbon source (Figure 2A). When glycerol was used up, the dissolved oxygen level increased rapidly, and the addition of methanol for induction was initiated at this time point. Samples for enzyme activity measurement and growth curve construction were taken at 12-h intervals and analyzed by SDS-PAGE. Maximal cell culture OD_600_ value (284) was obtained at 144 h; α-ABF activity (measured on WAX) in the supernatant medium was 67.1 U/mL, 3.6-fold higher than the value for shake flask culture (18.8 U/mL; Figures 1B, 2A). The maximal protein concentration in the supernatant was 0.9 mg/mL, and bands separated on SDS-PAGE were similar to those from the shake flask culture (Figures 1C, 2B).

Glycosylation, the most common post-translational modification occurring in proteins, strongly affects their conformation and function because of its complexity and variety (Taylor et al., 2009; Liu et al., 2022). Molecular weights derived from SDS-PAGE data (45–60 kDa) were notably higher than the theoretical value (35 kDa; Figures 1C, 2B). Bioinformatics analysis of TtAbf62 predicted one N-glycosylation site (Asn 108). Following deglycosylation, the recombinant protein appeared as a single 35 kDa band, consistent with the theoretical molecular weight (Figure 2C).

### 3.3. Enzymatic properties of TtAbf62

Enzymatic properties of recombinant TtAbf62 expressed by α-ABF were characterized using substrate WAX. Optimal reaction conditions were determined by evaluating the effects of pH (values ranging from 2.0 to 9.0) and temperature (values ranging from 20 to 80°C). Enzyme activity of TtAbf62 was maximal at pH 5.0 and 60°C and declined sharply at higher temperatures (Supplementary Figures 3A,B). When preincubated in the buffer of pH 5.0–7.0 for 1 h at 37°C, TtAbf62 retained >85% activity (Supplementary Figure 3C). Thermostability was maximal at 40°C; after 6 h incubation at this temperature, TtAbf62 retained ~85% activity (Supplementary Figure 3D).

Effects of various metal ions and chemical reagents on enzyme activity are summarized in Supplementary Table 2. TtAbf62 retained >50% activity after treatment with Ni^2+^, Co^2+^, Al^3+^, Mn^2+^, or Mg^2+^ and >85% activity after treatment with Na^+^, Zn^2+^, Ca^2+^, Fe^2+^, K^+^, Fe^3+^, EDTA, or Tween-20. The activity was strongly suppressed by Cu^2+^ (1.15% remained) and completely eliminated by SDS treatment.

Substrate specificities of TtAbf62 were determined by the standard DNS method (Table 1). The enzyme displayed the highest specific activities toward substrates WAX (179.07 U/mg) and RAX (191.14 U/mg); these values are greater than those reported, to date, for other ABFs from family GH62 (De La Mare et al., 2013; Wilkens et al., 2016). The specific activity of TtAbf62 toward *p*NPAf was much smaller (2.31 U/mg), and those toward substrates BWX, CMC, LBG, KGM, *p*NPX, *p*NPG, and *p*NPC were essentially zero.

**Table 1:** Substrate specificities of TtAbf62.

Substrate	Specific activity (U/mg)
Wheat arabinoxylan (WAX)	179.07 ± 6.68
Rye arabinoxylan (RAX)	191.14 ± 2.91
Birchwood xylan (BWX)	0.31 ± 0.02
Carboxymethyl cellulose sodium (CMC)	1.69 ± 0.06
Locust bean gum (LBG)	0.10 ± 0.02
Konjac glucomannan (KGM)	0.37 ± 0.003
4-nitrophenyl α-L-arabinofuranoside (*p*NPAf)	2.31 ± 0.04
4-nitrophenyl β-D-xylopyranoside (*p*NPX)	0.07 ± 0.002
4-nitrophenyl β-D-glucopyranoside (*p*NPG)	0.26 ± 0.01
4-nitrophenyl β-D-cellobioside (*p*NPC)	0.01 ± 0.001

Kinetic parameters of TtAbf62 were determined by varying concentrations of substrate WAX from 1 to 20 mg/mL (Supplementary Figure 4). Values obtained were K_m_ = 16.95 mg/mL and V_max_ = 198.41 U/mg.

### 3.4. Synergistic hydrolysis of barley/wheat by TtAbf62 in combination with other hemicellulases

**Figure 3:**
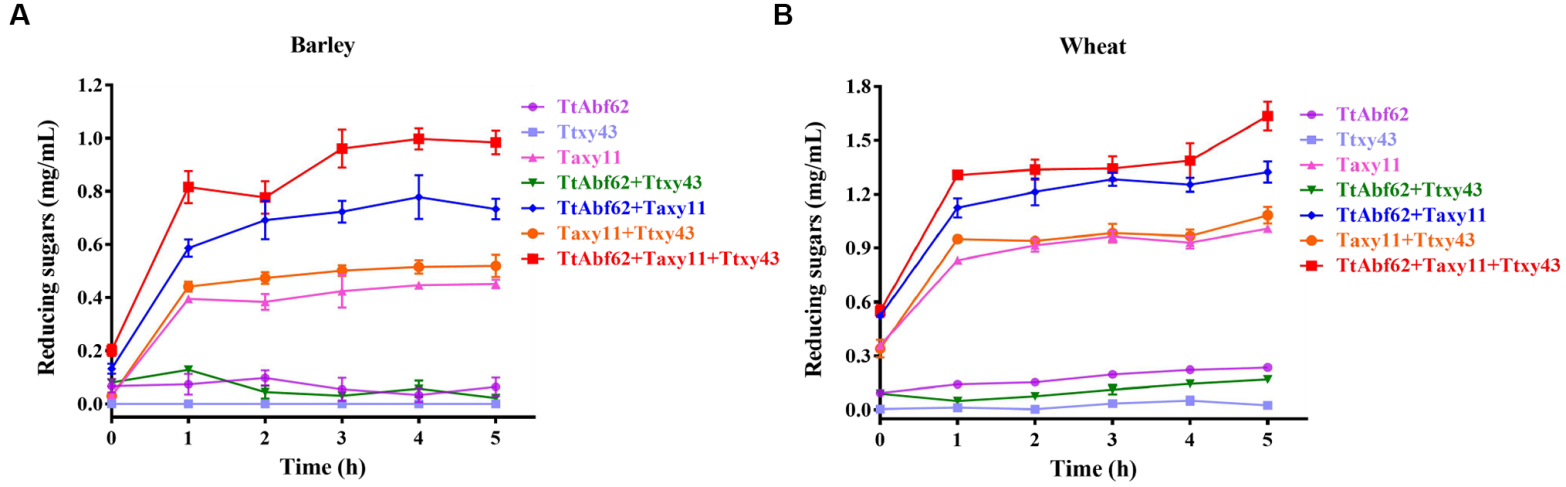
Synergistic hydrolytic activity of TtAbf62 in combination with hemicellulases. Release of reducing sugars from barley **(A)** and wheat **(B)** by ABF (TtAbf62), endoxylanase (Taxy11), and β-xylosidase (Ttxy43) individually and in combinations as indicated. Experiments were performed for 5 h at pH 6.0, temperature 45°C, enzyme concentration 50 U per g biomass, and substrate concentration 10% (w/v). Control: reaction system without loading enzyme solution.

In view of its high specific activity toward arabinoxylan, we evaluated the synergistic efficiency of TtAbf62 for barley/wheat hydrolysis. For the purpose of complete degradation, we combined TtAbf62 with two highly efficient hemicellulases (Taxy11 and Ttxy43), previously reported by Basit et al. (2019) and Zheng et al. (2020) (Figure 3; Supplementary Table 3). The activity of each enzyme was determined individually, enzymes in various combinations were incorporated in the reaction system at appropriate concentrations, and synergistic interactions were assayed at pH 6.0, 45°C (Figure 3; Supplementary Table 3).

TtAbf62 displayed no notable hydrolytic activity on barley/wheat. Therefore, a synergistic degradation approach (two or three hydrolases in combination) was applied to enhance hydrolysis. Hydrolytic efficiency was significantly increased when TtAbf62 and Taxy11 (xylanase) were reacted for 5 h. The combination of TtAbf62 and Ttxy43 displayed no notable synergistic interaction. However, the amount of reducing sugars was higher in the combination of both TtAbf62 and Taxy11, as compared with the combination of Taxy11 and Ttxy43.

The combination of TtAbf62/Taxy11/Ttxy43 displayed stronger synergistic interaction than that of the combination of two enzymes. The release of reducing sugars was significantly higher for the three-enzyme combination relative to the sum of single-enzyme treatments: 85.71% for barley hydrolysis and 33.33% for wheat hydrolysis (Figure 3; Supplementary Table 3). These findings demonstrate the ability of “multienzyme cocktails” to strongly increase the hydrolysis of biomass through synergistic interaction, in comparison with single-enzyme or two-enzyme treatments.

### 3.5. Construction of TtAbf62 mutants by site-directed mutagenesis and its catalytic mechanism

**Figure 4:**
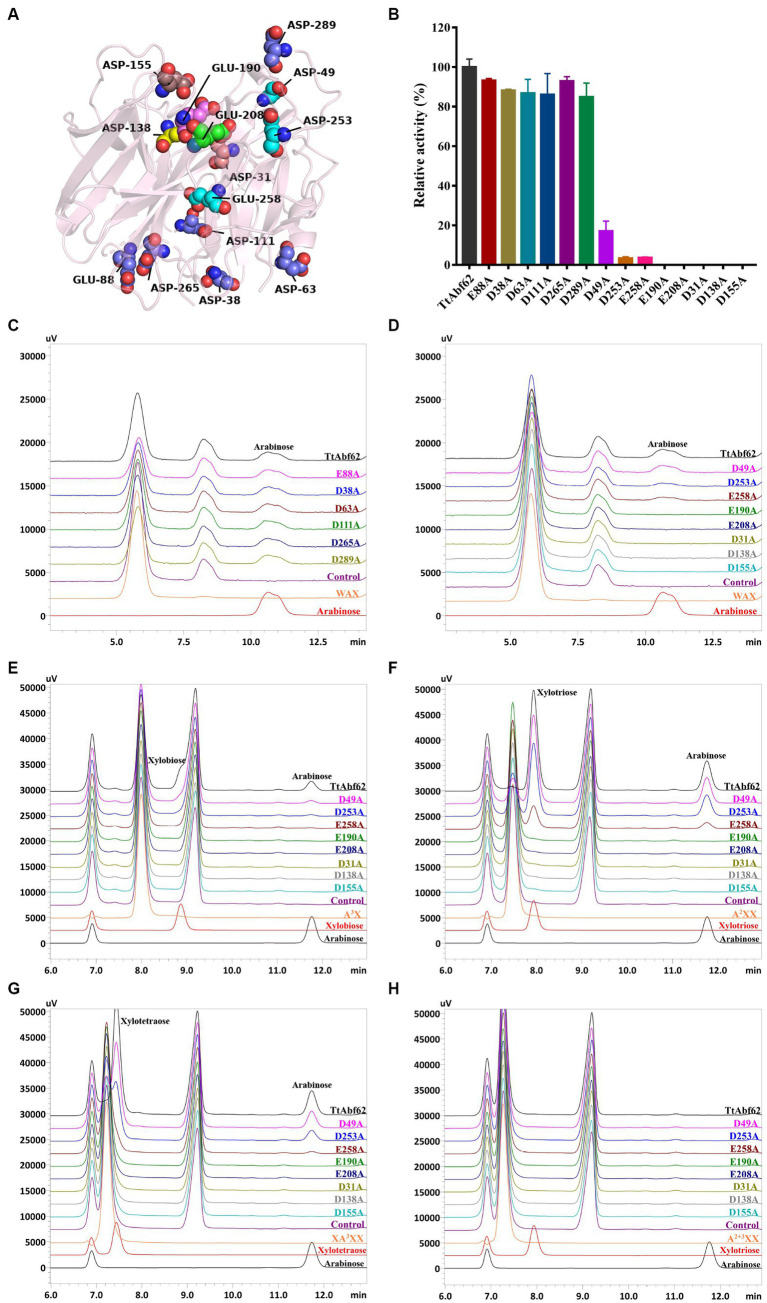
Construction of TtAbf62 mutants. **(A)** Protein 3D structure of TtAbf62, from software programs SWISS-MODEL and PyMOL. Presumed catalytic residues are labeled. **(B)** Enzyme activity levels of TtAbf62 and constructed mutant strains. **(C,D)** HPLC analysis of wheat arabinoxylan hydrolysis products of TtAbf62 and its mutants (peaks at 10.5–11.5 min indicate the arabinose, and the peak at 8.0–8.5 min is an unknown component of the reaction system). **(E–H)** HPLC analysis of hydrolysates of arabinoxylo-oligosaccharides. Hydrolysis of **(E)** A^3^X, **(F)** A^2^XX, **(G)** XA^3^XX, and **(H)** A^2 + 3^XX by TtAbf62 and its mutants; catalytic products were analyzed by HPLC (peaks at 11.8 min indicate the arabinose, and the peaks at 6.9 and 9.2 min are different unknown components of the reaction system). Control, reaction system containing inactivated enzyme solution.

The role of TtAbf62 in promoting arabinoxylan degradation was elucidated by substituting relevant residues with alanine, investigating the role of catalytic residues towards substrate recognition and catalysis in mutants (Figure 4A). The mutant strains were induced for expression of recombinant proteins and SDS-PAGE analysis indicated the molecular weight of ~45–60 kDa for recombinant proteins, consistent with values for TtAbf62 (Supplementary Figure 5). Mutant strains E88A, D38A, D63A, D111A, D265A, and D289A displayed catalytic activity toward WAX, similar to that of TtAbf62 (Figure 4B). In contrast, mutants E190A, E208A, D31A, D138A, and D155A showed loss of activity toward WAX, and catalytic activities of mutants D49A, D253A, and E258A were, respectively, 83%, 97%, and 96% lower than that of TtAbf62 (Figure 4B). HPLC analysis showed that alanine substitution of Glu^88^, Asp^38^, Asp^63^, Asp^111^, Asp^265^, and Asp^289^ still resulted in arabinose production, whereas substitution of Glu^190^, Glu^208^, Asp^31^, Asp^138^, and Asp^155^ yielded no arabinose production (Figures 4C,D). Mutants D49A, D253A, and E258A liberated trace amounts of arabinose in WAX degradation (Figure 4D). TtAbf62 displayed characteristic ABF activity, i.e., released exclusively arabinose from WAX. These findings, taken together, indicate that Glu^190^, Glu^208^, Asp^31^, Asp^138^, and Asp^155^ are the essential catalytic sites for TtAbf62 activity and that Asp^49^, Asp^253^, and Glu^258^ have auxiliary catalytic roles in side chain removal. The catalytic sites Glu^188^, Asp^28^, and Asp^136^ that B. Svensson’s group showed to be conserved in AnAbf62A-m2,3 (Wilkens et al., 2016) are also conserved in TtAbf62, in which they correspond to Glu^190^, Asp^31^, and Asp^138^ (Supplementary Figure 1).

A^3^X, A^2^XX, A^2 + 3^XX, and XA^3^XX (see section 2.8) were used as substrates for investigating the catalytic mechanism of TtAbf62 and the functions of active catalytic sites in the removal of side chains from arabinoxylan. HPLC analysis showed that TtAbf62 was able to release arabinose from single-arabinofuranosyl arabinoxylo-oligosaccharides substrates (A^3^X, A^2^XX, and XA^3^XX) but not from double-arabinofuranosyl substrate (A^2 + 3^XX; Figures 4E–H; the differences in arabinose peak between the two series of chromatograms are due to the use of different chromatographic columns, as shown in Figures 4C,D and Figures 4E–H). E190A, E208A, D31A, D138A, and D155A displayed no activity against any of the four substrates, indicating that the catalytic sites Glu^190^, Glu^208^, Asp^31^, Asp^138^, and Asp^155^ were involved in substrate hydrolysis. The amount of arabinose released by hydrolysis of A^3^X was significantly lower for D49A and D253A than for TtAbf62. Asp^49^ and Asp^253^ evidently played auxiliary roles in the removal of arabinofuranosyl monosubstitution from the linear xylose backbone (X < 3; Figure 4E). E258A released trace amounts of arabinose through the hydrolysis of A^2^XX and XA^3^XX, indicating that Glu^258^ played an important auxiliary role in removing arabinofuranosyl side chains from the linear xylose backbone (X ≥ 3; Figures 4F,G).

## 4. Discussion

α-L-arabinofuranosidases (ABFs) have potential economic and environmental importance based on complete bioconversion of hemicellulose for biofuel production and synthesis of various biochemical products (Poria et al., 2020). Numerous ABFs from different microorganisms have been isolated and characterized. Despite their wide range of potential industrial applications, ABFs have received relatively little attention due to the lack of high-yield strains.

Therefore, here, we described the identification of ABF (TtAbf62) from *T. thermophilus* which displayed high-enzyme yield and activity when expressed in *P. pastoris*. Codon optimization and signal peptide modification strategies were used to optimize TtAbf62 expression. Extracellular secretion of the enzyme was strongly affected by the use of an appropriate signal peptide. Three engineered recombinant strains were constructed based on a codon-optimized gene, and the highest extracellular ABF activity (18.8 U/mL) was obtained for one of the strains (Figures 1A,B). The choice of the signal peptide is a key factor in controlling the secretion of recombinant protein, and α-factor signal is an excellent signal peptide for extracellular TtAbf62 production (Miao et al., 2021b).

High-density fermentation has shown an increase in yield and activity of recombinant cellulases and hemicellulases (Zheng et al., 2020; Miao et al., 2021a). However, limited literature is available regarding high-density fermentation for recombinant ABF production, particularly for family GH62. In this study, increased production of the TtAbf62 enzyme (~3.6-fold) was achieved through high-density fermentation (Figure 2A). The corresponding specific activity (74.55 U/mg) was lower than the mentioned values in Table 1 (179.07 U/mg); however, a large number of intracellular proteins were released by cell lysis during the late fermentation process, resulting in a higher protein concentration and a lower specific activity value. In addition, TtAbf62 showed attractive enzyme properties. Mostly, commercial ABFs have maximal activity at 40°C and pH range 6.0–7.5; however, TtAbf62 displayed good thermostability and pH stability at approximately 50°C and pH 5.0–7.5 (Supplementary Figure 3C and D). In contrast, the stability of TtAbf62 at high temperatures is favorable for catalysis of arabinoxylan, debranching in the initial stage of the industrial malt crushing process (for enzyme activation: 37°C/30 min; for protein degradation: 45°C–55°C/30–60 min). TtAbf62 also displays good flexibility and stability in moderately acidic environments, which is advantageous in industrial processing. Metal ions in wort (liquid extracted from the mashing process during the brewing of beer or whisky) originating from brewing water and malt are primarily Na^+^, Mg^2+^, Fe^3+^, and Ca^2+^ (Li et al., 2020). TtAbf62 activity was slightly affected by these ions with no inhibition by EDTA (Supplementary Table 2), suggesting that cofactors are not required for its catalytic activity. TtAbf62, thus, has the potential advantages of stable fermentation activity and low cost during the brewing process.

The enzyme activity of TtAbf62 toward natural substrates (WAX and RAX) is highly efficient. In particular, the specific activity toward low-viscosity WAX (WAX-LV) is the highest of any characterized GH62 enzymes and promotes the efficiency of saccharification of hemicellulosic biomass (Wilkens et al., 2017). *p*NPAf has been reported to be a good substrate for ABFs of other GH families (Lagaert et al., 2014). However, TtAbf62 and some other GH62 ABFs display low activity toward *p*NPAf, suggesting that TtAbf62 is a “type B” ABF, characterized by preferential activity/specificity toward polysaccharides (Saha, 2000). In contrast to GH43 ABFs, GH62 ABFs do not affect the β-1,4-Xylp linkages (McKee et al., 2012).

Arabinoxylan is a major structural component of cell walls in various barley grain tissues (Izydorczyk and Dexter, 2008) and comprises ~70% of starchy endosperm cell wall polysaccharides in wheat (Pellny et al., 2012). Improvement of its bioconversion efficiency in agricultural waste by-products and production in functional foods is therefore highly desirable (Lin et al., 2021). Substrates with a high content of arabinofuranosyl residues are not easily degraded by hydrolases without the participation of ABFs. A complete breakdown of complex substances containing arabinoxylan as a major component requires various hemicellulases, particularly ABFs, xylanases, and xylosidases. ABFs are important accessory enzymes necessary for the complete hydrolysis of WAX (Saha, 2000). Therefore, TtAbf62 was combined with hemicellulases (Taxy11 and Ttxy43), previously reported by our group (Basit et al., 2019; Zheng et al., 2020). Arabinoxylan hydrolysis efficiency was significantly increased with the highest yield of reducing sugars by following the initial optimization of all reaction conditions for synergism (Figure 3; Supplementary Table 3). Taxy11 acts as an endoxylanase catalyzing hydrolysis of xylan backbone, and synergistic activity of ABFs resulted from the removal of arabinofuranosyl side chains from arabinoxylan and increased accessibility of xylanase to the substrate. The use of Taxy11/Ttxy43 combination enhanced the catalysis of insoluble substrates, and xylosidase suppressed inhibition of xylanase by hydrolyzing intermediate xylooligosaccharides (Figure 3; Supplementary Table 3). Data shown in Supplementary Table 3 suggest that the level of reducing sugars was more strongly increased by the TtAbf62/Taxy11 combination than by the Taxy11/Ttxy43 combination. This apparent discrepancy is probably due to the involvement of ABFs in the rate-limiting step of the WAX degradation process during the synergistic reaction (Laothanachareon et al., 2015). This degradation process is more strongly affected by the removal of side chains than the hydrolysis of intermediate product arabinoxylooligomers by xylosidases. ABFs, in combination with other hydrolases, have been shown to enhance enzymatic bioconversion of lignocellulosic biomass, and hydrolytic efficiency is promoted by the synergistic activity of ABFs and other biomass-degrading enzymes (Delabona et al., 2013). The enzymatic hydrolysis yield of wheat straw was similarly increased by using a mixture of ABF, endoxylanase, and cellulase (Alvira et al., 2011).

**Figure 5:**
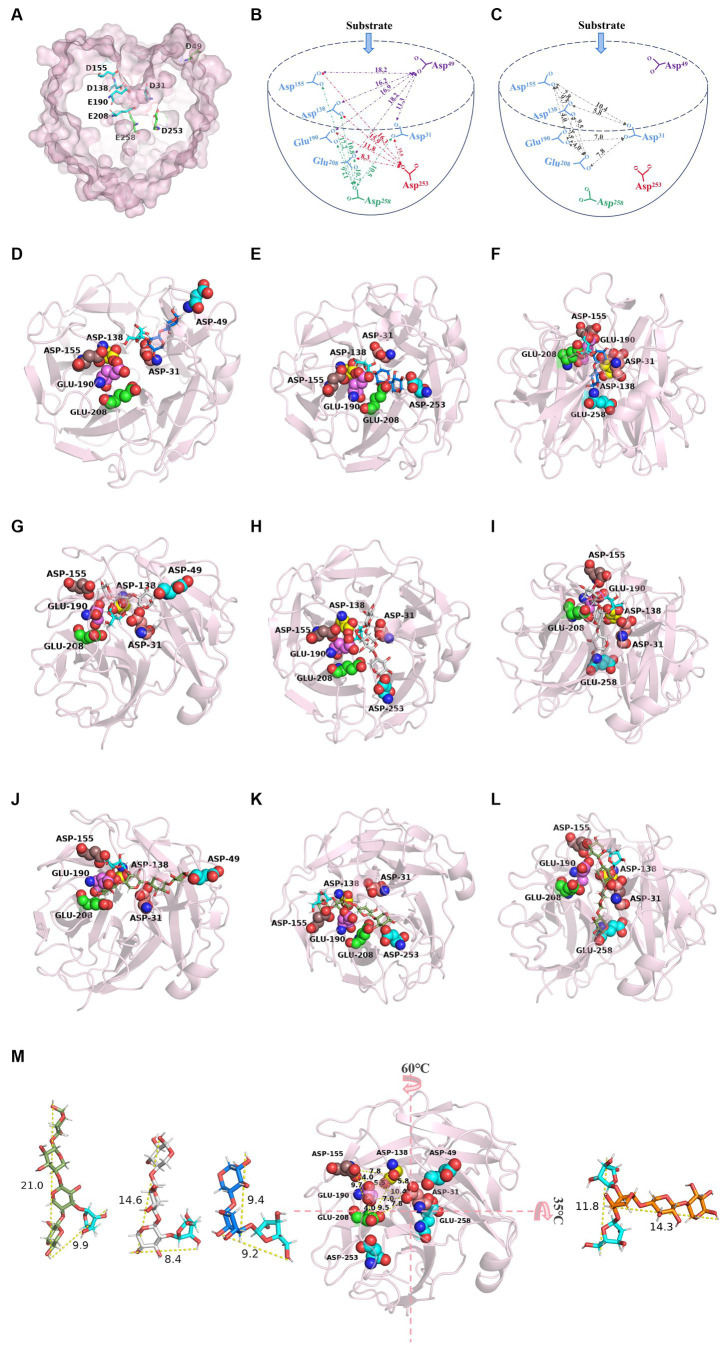
Catalytic pattern of TtAbf62. **(A)** Surface representation of TtAbf62 showing catalytic residues in proposed catalytic pocket. **(B,C)** Schematic diagram of catalytic region, reflecting distances (Å) among catalytic sites. Enzyme–substrate binding structural interaction analyses showed structure of TtAbf62 (pink) superimposed with **(D–F)** A^3^X, **(G–I)** A^2^XX, and **(J–L)** XA^3^XX. **(M)** Diameters (Å) of xylooligosaccharides and PyMOL analysis (vertical view) of distances (Å) among catalytic sites, such as Glu^190^, Glu^208^, Asp^31^, Asp^138^, and Asp^155^. A^3^X (dark blue, xylobiose; cyan, arabinofuranosyl), A^2^XX (gray, xylotriose; cyan, arabinofuranosyl), XA^3^XX (green, xylotetraose; cyan, arabinofuranosyl), A^2 + 3^XX (orange, xylotriose; cyan, arabinofuranosyl). Catalytic residues are represented as spheres: Glu^190^ (pink), Glu^208^ (green), Asp^31^ (salmon), Asp^138^ (yellow), Asp^155^ (brown), Asp^49^ (cyan), Asp^253^ (cyan), and Glu^258^ (cyan).

In this study, two software programs, namely, SWISS-MODEL and AlphaFold (2.2.0), were utilized to predict the structure of TtAbf62. The generated models had high quality based on SWISS-MODEL parameters. The prediction model, with a sequence similarity of 75.75% to the template (SthAraf62A, PDB ID: 4O8N; Wang et al., 2014), had the highest Global Model Quality Estimation (GMQE) value of 0.93 and a good Qualitative Model Energy Analysis (QMEAN) value of 0.89. Comparison between the model structure predicted by homology modeling, and AlphaFold (2.2.0) revealed a root-mean-square deviation (RMSD) of 0.359 Å (Supplementary Figure 6), indicating that the model predicted by homology modeling is almost as valid as that predicted by AlphaFold (2.2.0). Family GH62 belongs to the same GH clan (F) as does family GH43 (Lagaert et al., 2014). 3D structure prediction model of TtAbf62 shows a general folding pattern representing a five-bladed β-propeller, in which each blade has four twisted antiparallel β-strands of varying length, oriented radially around a pseudo-5-fold axis (Figure 4A; Wilkens et al., 2017). Catalytic residues are situated on the surface of the catalytic pocket and constitute a hemispherical catalytic space located in the central depression formed by the five blades (Figures 5A–C).

HPLC analysis revealed the specificity of TtAbf62 in the removal of arabinofuranosyl side chains linked in positions C-2 and C-3 of monosubstituted xylose residues rather than double substitution from arabinoxylo-oligosaccharides (AXOS; Figures 4E–H). Consistently with this finding, previous studies indicate that almost all characterized GH62 ABFs display AXH-m2,3 activity, which affects only monosubstituted (C-2 or C-3) xylopyranosyl units in arabinoxylan (Phuengmaung et al., 2018). Examples of such ABFs include PfAbf62 (De La Mare et al., 2013), SWUAbf62A (Phuengmaung et al., 2018), and SthAbf62A (Wang et al., 2014). We described the catalytic pattern of the TtAbf62 enzyme on arabinoxylan in barley/wheat by analyzing its hydrolytic phenotype on arabinoxylan and AXOS, using previously developed strategies of site-directed mutation of key amino acids in enzyme active center, and interactional analyses of enzyme-substrate binding structures. Our speculation is that substrates capable of entering the catalytic pocket exhibit efficient degradation of the side chain. Conversely, substrates that are unable to enter the pocket are unable to remove the side chain. The PyMOL measurement wizard indicates that the side chain and backbone length of A^3^X, A^2^XX, XA^3^XX, and A^2 + 3^XX are approximately 9.2, 8.4, 9.9, and 11.8 Å and 9.4, 14.6, 21, and 14.3 Å, respectively. When Asp^49^ recognized the reducing end of A^3^X, the xylose residue from the reducing end on the main chain located at the entrance of the catalytic pocket facilitates the entrance of arabinofuranosyl side chain at the center of the catalytic pocket for debranching reaction. When the reducing end bound to Asp^253^ and Glu^258^, the xylobiose backbone (9.4 Å) was intercalated between Asp^253^-Glu^190^ and Glu^258^-Asp^31^ (respective distances 11.8 Å and 10.5 Å), facilitating degradation of the side chain through joint activity of key catalytic sites (Figures 5B,D–F,M). When the reducing end of A^2^XX bound to Asp^49^, Asp^253^, and Glu^258^, its non-reducing end of the side chain substitution faced toward the bottom of the catalytic pocket, and the xylotriose backbone was embedded in the cleft between the active sites (Figures 5G–I). In contrast, when the reducing end of XA^3^XX bound to Asp^49^, Asp^253^, and Glu^258^, its arabinofuranosyl side chain faced toward the opening of the catalytic pocket, and the xylotetraose backbone was inserted in the gap between the catalytic sites, facilitating removal of arabinofuranosyl monosubstitutions by TtAbf62 (Figures 5J–L). Distance between double-arabinofuranosyl groups of A^2 + 3^XX was found to be 11.8 Å, and distances between the five essential catalytic sites ranged from 4.0 to 10.4 Å. Consequent steric interference presumably prevented substrate binding to the enzyme active center, in such a way that A^2 + 3^XX could not reach the center of the catalytic pocket, and TtAbf62 displayed no catalytic activity for this substrate (Figure 5M). TtAbf62 displayed hydrolytic activity toward monosubstituted AXOS; however, the active amino acid residues formed different xylan backbone-binding clefts on the catalytic domain, and this variability resulted in differing topologies of TtAbf62 with A^3^X/A^2^XX/XA^3^XX-binding clefts (Figures 5D–L). A^2 + 3^XX did not fit into the binding cleft of TtAbf62 because of spatial obstruction (Figure 5M), and thus, TtAbf62 did not hydrolyze A^2 + 3^XX (Figure 4H). In view of the complex structure of arabinoxylan, extensive further studies are needed to elucidate the detailed mechanism of its hydrolysis by TtAbf62.

## 5. Conclusion

A novel α-L-arabinofuranosidase (ABF) from *T. thermophilus* (termed as TtAbf62) was characterized, and its enzyme activity was enhanced by signal peptide modification strategy and optimized by scaling up fermentation. The maximal specific activity of TtAbf62 on substrate WAX was higher than those of any other GH62 ABF characterized, to date, and 2.7-fold higher than that recently reported for AnAbf62A-m2,3, which showed maximal specific activity (67 U/mg) on WAX-LV (Wilkens et al., 2016). Site-directed mutagenesis and enzyme-substrate binding structural interaction analyses of TtAbf62 clarified its mechanism of hydrolysis of arabinoxylo-oligosaccharides in AXH-m2,3 activity mode. TtAbf62 displayed clear synergistic activity with Taxy11 and Ttxy43 in barley/wheat hydrolysis, indicating its capability to effectively enhance the saccharification of arabinoxylan in hemicellulose. The diverse beneficial enzymatic properties of TtAbf62, as elucidated here, make it a strong candidate for development and application in many industrial processes involving arabinoxylan degradation and biomass conversion.

## Data availability statement

The original contributions presented in the study are included in the article/supplementary material, further inquiries can be directed to the corresponding author.

## Author contributions

JW: formal analysis, investigation, data curation, and writing—original draft. TM, QL, ST, SC, NA, HW, YW, and FZ: formal analysis and investigation. AB: writing—reviewing and editing. WJ: funding acquisition, formal analysis, resources, project administration, supervision, and writing—reviewing and editing. All authors contributed to the article and approved the submitted version.

## Glossary

**Table:** 

ABFs	α-L-arabinofuranosidases
AXHs	Arabinoxylan-arabinofuranohydrolases
GH	Glycoside hydrolase
CAZy	Carbohydrate-active enzymes
WAX	Wheat arabinoxylan
WAX-LV	Wheat arabinoxylan low-viscosity
RAX	Rye arabinoxylan
BWX	Beechwood xylan
A^3^X	3^2^-α-L-arabinofuranosyl-xylobiose
A^2^XX	2^3^-α-L-arabinofuranosyl-xylotriose
A^2 + 3^XX	2^3^,3^3^-di-α-L-arabinofuranosyl-xylotriose
XA^3^XX	3^3^-α-L-arabinofuranosyl-xylotetraose
CMC	Sodium carboxymethyl cellulose
LBG	Locust bean gum
*p*NPAf	4-nitrophenyl α-L-arabinofuranoside
*p*NPX	4-nitrophenyl β-D-xylopyranoside
*p*NPG	4-nitrophenyl β-D-glucopyranoside
*p*NPC	4-nitrophenyl β-D-cellobioside
KGM	Konjac glucomannan
YPDS	Agar plates coated with yeast extract peptone dextrose with sorbitol
BMMY	Buffered minimal methanol medium
DNS	3,5-dinitrosalicylic acid
*p*NP	p-nitrophenol
BSM	Basal salt medium
Endo H	Endoglycosidase H
AXOS	Arabinoxylo-oligosaccharides
GMQE	Global model quality estimation
QMEAN	Qualitative model energy analysis
RMSD	Root-mean-square deviation

## Footnotes

^1^ https://services.healthtech.dtu.dk/services/NetAcet-1.0/

^2^ https://swissmodel.expasy.org

^3^ http://www.pymol.org

^4^ www.chemspider.com/

^5^ http://www.clustal.org/omega/

^6^ https://www.jalview.org

^7^ http://www.ncbi.nlm.nih.gov/BLAST/

^8^ http://www.cazy.org/

^9^ https://web.expasy.org

^10^ https://services.healthtech.dtu.dk/services/SignalP-5.0/
