# Supplementary material for: Highly efficient synergistic activity of an α-L-arabinofuranosidase for degradation of arabinoxylan in barley/wheat
Source: Front Microbiol. 2023 Nov 3;14:1230738. doi: 10.3389/fmicb.2023.1230738 (PMC10655120; doi:10.3389/fmicb.2023.1230738)
Supplement: Supplementary file 1 [file Image_1.pdf]

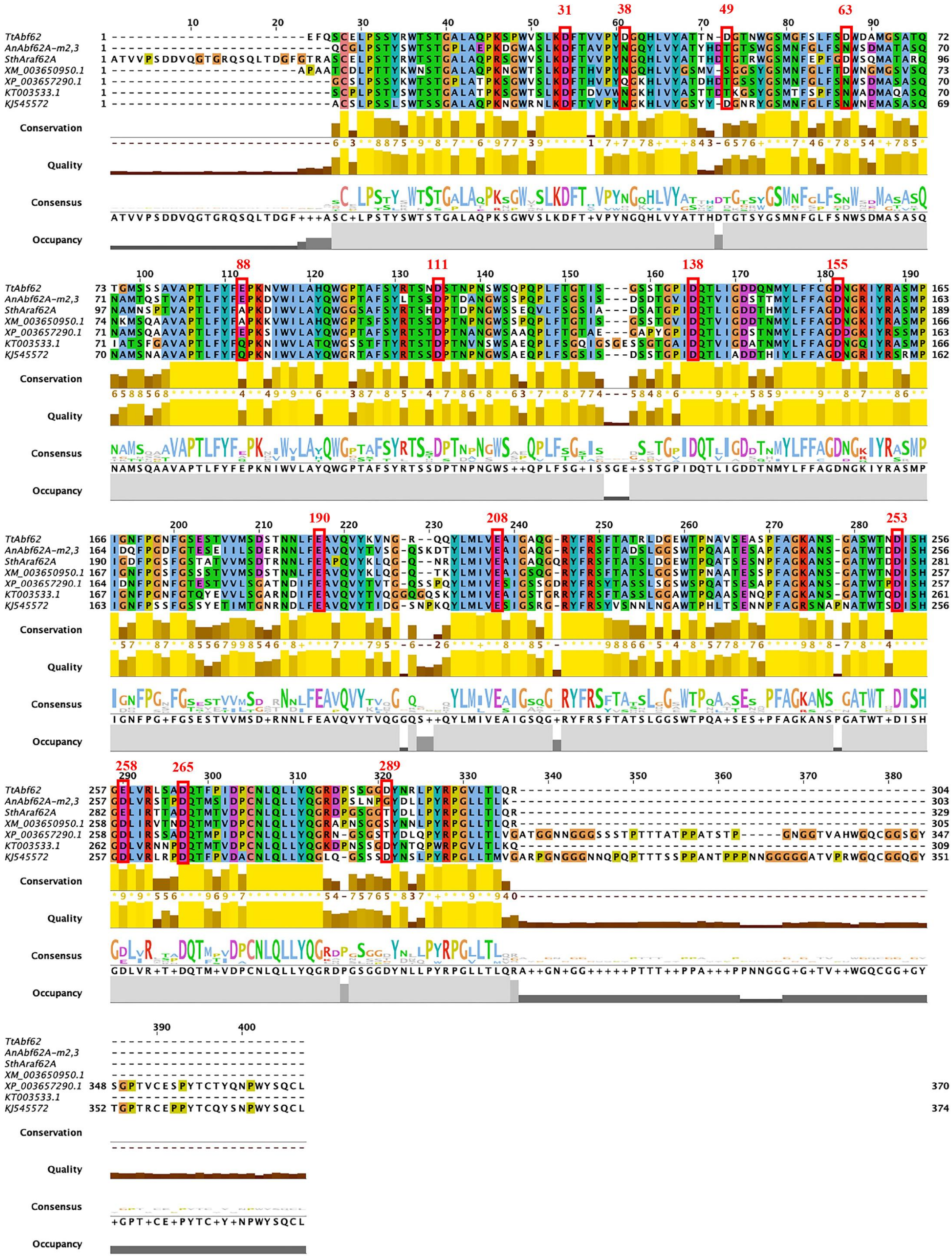

Figure 1. **Multiple-sequence alignment of TtAbf62 with other GH62 α-L-arabinofuranosidases (excluding the signal peptide).** The provided graphs illustrate conservation, quality, consensus and occupancy. Residues Glu[88], Glu[190], Glu[208], Glu[258], Asp[31], Asp[38], Asp[49], Asp[63], Asp[111], Asp[138], Asp[155], Asp[253], Asp[265], and Asp[289] (order number in TtAbf62) which may participate in the catalytic process are highlighted in red. Positions that are fully conserved in the conservation histogram are indicated with an asterisk, and columns that retain all properties are marked with a plus. The size of the amino acid symbol in the consensus status represents the consistency with the conservation of the residues.
