# Supplementary material for: Highly efficient synergistic activity of an α-L-arabinofuranosidase for degradation of arabinoxylan in barley/wheat
Source: Front Microbiol. 2023 Nov 3;14:1230738. doi: 10.3389/fmicb.2023.1230738 (PMC10655120; doi:10.3389/fmicb.2023.1230738)
Supplement: Supplementary file 2 [file Image_2.pdf]

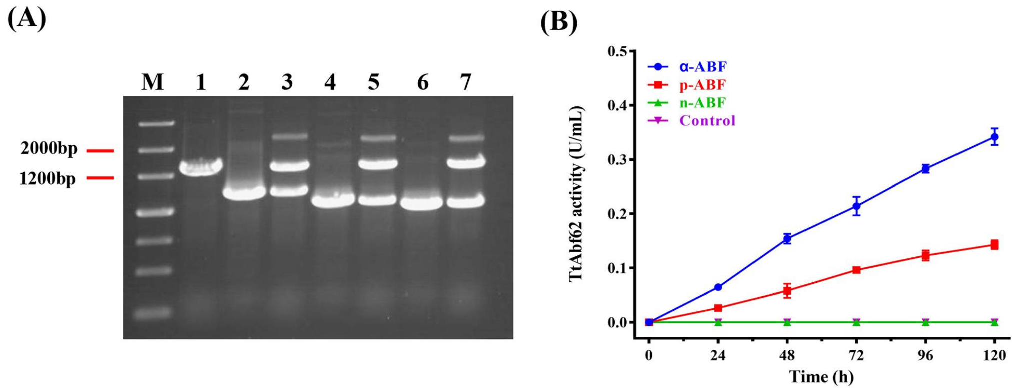

Figure 2. **PCR verification of the constructed engineered strains. (A)** PCR primers AOX-F/AOX-R were used to identify positive constructs and control (X-33) strain (without target gene). M, DNA markers; 1, control (X-33) strain; 2, plasmid pPICZα-*oabf*; 3, engineered strain α-ABF; 4, plasmid pPICZp-*oabf*; 5, engineered strain p-ABF; 6, plasmid pPICZ-*oabf*; 7, engineered strain n-ABF. **(B)** Comparison of α-ABF, p-ABF, n-ABF, and control (X-33) strains in terms of TtAbf62 activity levels toward *p*NPAf. Assays were performed in triplicate.
