# Supplementary material for: Highly efficient synergistic activity of an α-L-arabinofuranosidase for degradation of arabinoxylan in barley/wheat
Source: Front Microbiol. 2023 Nov 3;14:1230738. doi: 10.3389/fmicb.2023.1230738 (PMC10655120; doi:10.3389/fmicb.2023.1230738)
Supplement: Supplementary file 3 [file Image_3.pdf]

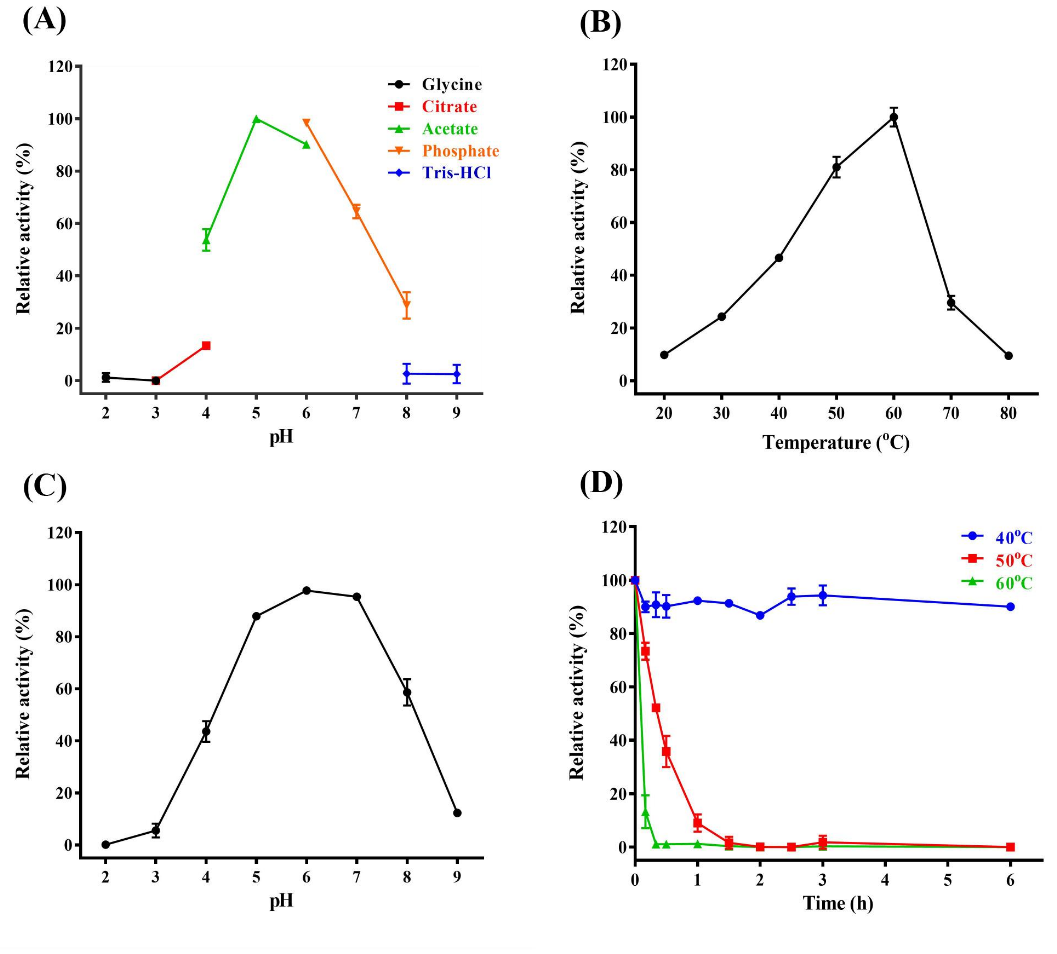

Figure 3. <u>Enzymatic properties of TtAbf62 in α-ABF</u>. (A) Effect of pH on TtAbf62 activity. **(B)** Effect of temperature on TtAbf62 activity. **(C)** pH-stability of TtAbf62 after 1 h incubation at 37°C. **(D)** Thermo-stability of TtAbf62 at optimal pH.
