# Supplementary material for: Highly efficient synergistic activity of an α-L-arabinofuranosidase for degradation of arabinoxylan in barley/wheat
Source: Front Microbiol. 2023 Nov 3;14:1230738. doi: 10.3389/fmicb.2023.1230738 (PMC10655120; doi:10.3389/fmicb.2023.1230738)
Supplement: Supplementary file 4 [file Image_4.pdf]

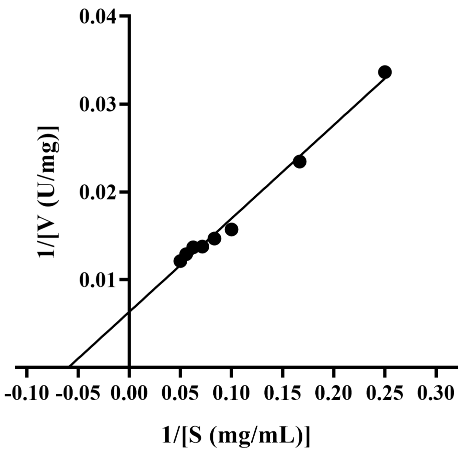

Figure 4. **Linear regression curves on Lineweaver-Burk double inverse plots to determine the kinetic parameters of TtAbf62.**
