# Supplementary material for: Highly efficient synergistic activity of an α-L-arabinofuranosidase for degradation of arabinoxylan in barley/wheat
Source: Front Microbiol. 2023 Nov 3;14:1230738. doi: 10.3389/fmicb.2023.1230738 (PMC10655120; doi:10.3389/fmicb.2023.1230738)
Supplement: Supplementary file 5 [file Image_5.pdf]

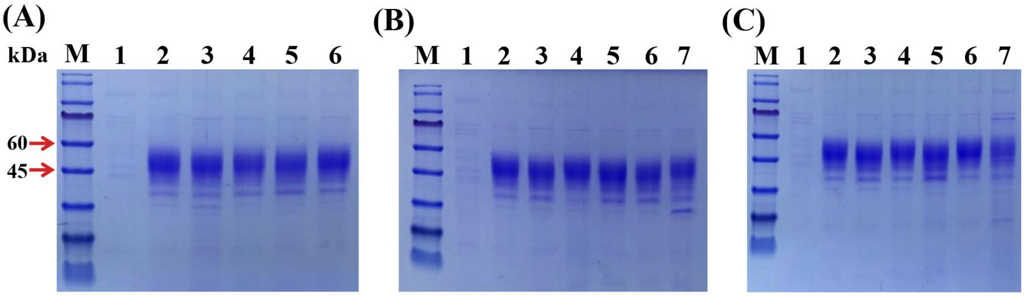

Figure 5. <u>**SDS-PAGE analysis of TtAbf62 and its mutants**</u>**.** **(A)** M, molecular marker; 1, control (X-33); 2, TtAbf62; 3, E88A; 4, E190A; 5, E208A; 6, E258A. **(B)** M, molecular marker; 1, control (X-33); 2, TtAbf62; 3, D31A; 4, D38A; 5, D49A; 6, D63A; 7, D111A. **(C)** M, molecular marker; 1, control (X-33); 2, TtAbf62; 3, D138A; 4, D155A; 5, D253A; 6, D265A; 7, D289A.
