# Supplementary material for: Highly efficient synergistic activity of an α-L-arabinofuranosidase for degradation of arabinoxylan in barley/wheat
Source: Front Microbiol. 2023 Nov 3;14:1230738. doi: 10.3389/fmicb.2023.1230738 (PMC10655120; doi:10.3389/fmicb.2023.1230738)
Supplement: Supplementary file 6 [file Image_6.pdf]

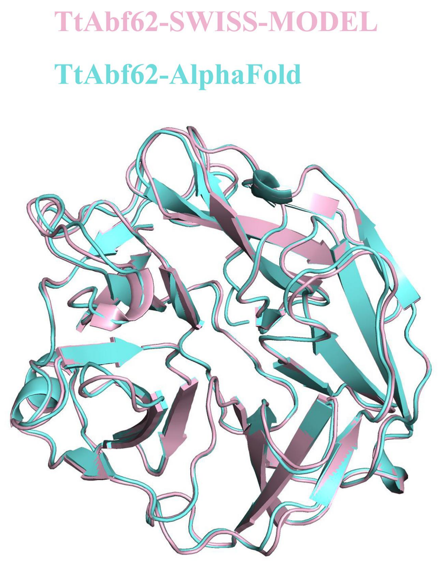

Figure 6. **Prediction modeling of TtAbf62 by SWISS-MODEL (shown in pink) and AlphaFold 2.2.0 (shown in cyan).**
