# Supplementary material for: Highly efficient synergistic activity of an α-L-arabinofuranosidase for degradation of arabinoxylan in barley/wheat
Source: Front Microbiol. 2023 Nov 3;14:1230738. doi: 10.3389/fmicb.2023.1230738 (PMC10655120; doi:10.3389/fmicb.2023.1230738)
Supplement: Supplementary file 7 [file Table_1.docx]

**Table 1.** Primers used, and their sequences.

| **Primers** | **Sequence (5’-3’)** |
| --- | --- |
| **pPICZ-*oabf*-F** | **CGGAATTCCAATCTTGTGAGTTGCCATCTTCT** |
| **pPICZ-*oabf*-R** | **GGGGTACCTTATCTTTGCAAAGTCAAAACACC** |
| **pPICZp-*oabf*-F** | **CGGAATTCATGAGACCAACTAGACCAGGTATT** |
| **pPICZp-*oabf*-R** | **GGGGTACCTTATCTTTGCAAAGTCAAAACACC** |
| **AOX-F** | **GACTGGTTCCAATTGACAAGC** |
| **AOX-R** | **GCAAATGGCATTCTGACATCC** |
| **E88A-F** | **TACTTCGCTCCAAAGAACGTTTGGATTTTGGCTCACCAAT** |
| **E88A-R** | **CTTTGGAGCGAAGTAGAACAAAGTTGGAGCAACAGCAGAA** |
| **E190A-F** | **TTGTTCGCTGCTGTTCAAGTTTACAAGGTTAACGGTAGAC** |
| **E190A-R** | **AACAGCAGCGAACAAGTTGTTAGTAGAGTCAGACATAACA** |
| **E208A-F** | **TTGGTTGCTGCTATTGGTGCTCAAGGTAGATACTTCAGAT** |
| **E208A-R** | **AATAGCAGCAACCAACATCAAGTATTGTTGTCTACCGTTA** |
| **E258A-F** | **CACGGTGCTTTGGTTAGATTGTCTGCTGACCAAACTTTCC** |
| **E258A-R** | **AACCAAAGCACCGTGAGAAATGTCGTTAGTCCAAGAAGCA** |
| **D31A-F** | **TTGAAGGCTTTCACTGTTGTTCCATACGACGGTCAACACT** |
| **D31A-R** | **AGTGAAAGCCTTCAAAGAAACCCATGGAGACTTTGGTTGA** |
| **D38A-F** | **CCATACGCTGGTCAACACTTGGTTTACGCTACTACTAACG** |
| **D38A-R** | **TTGACCAGCGTATGGAACAACAGTGAAGTCCTTCAAAGAA** |
| **D49A-F** | **ACTAACGCTGGTACTAACTGGGGTTCTATGGGTTTCTCTT** |
| **D49A-R** | **AGTACCAGCGTTAGTAGTAGCGTAAACCAAGTGTTGACCG** |
| **D63A-F** | **TTCTCTGCTTGGGACGCTATGGGTTCTGCTACTCAAACTG** |
| **D63A-R** | **GTCCCAAGCAGAGAACAAAGAGAAACCCATAGAACCCCAG** |
| **D111A-F** | **TCTAACGCTTCTACTAACCCAAACTCTTGGTCTCAACCAC** |
| **D111A-R** | **AGTAGAAGCGTTAGAAGTTCTGTAAGAGAAAGCAGTTGGA** |
| **D138A-F** | **CCAATTGCTCAAACTTTGATTGGTGACGACCAAAACATGT** |
| **D138A-R** | **AGTTTGAGCAATTGGACCAGTAGAAGAACCAGAAATAGTA** |
| **D155A-F** | **TGTGGTGCTAACGGTAAGATTTACAGAGCTTCTATGCCAA** |
| **D155A-R** | **ACCGTTAGCACCACAGAAGAACAAGTACATGTTTTGGTCG** |
| **D253A-F** | **ACTAACGCTATTTCTCACGGTGAGTTGGTTAGATTGTCTG** |
| **D253A-R** | **AGAAATAGCGTTAGTCCAAGAAGCACCAGAGTTAGCCTTA** |
| **D265A-F** | **TCTGCTGCTCAAACTTTCCCAATTGACCCATGTAACTTGC** |
| **D265A-R** | **AGTTTGAGCAGCAGACAATCTAACCAACTCACCGTGAGAA** |
| **D289A-F** | **GGTGGTGCTTACAACAGATTGCCATACAGACCAGGTGTTT** |
| **D289A-R** | **GTTGTAAGCACCACCAGAAGATGGGTCTCTACCTTGGTAC** |
