# Supplementary material for: Highly efficient synergistic activity of an α-L-arabinofuranosidase for degradation of arabinoxylan in barley/wheat
Source: Front Microbiol. 2023 Nov 3;14:1230738. doi: 10.3389/fmicb.2023.1230738 (PMC10655120; doi:10.3389/fmicb.2023.1230738)
Supplement: Supplementary file 8 [file Table_2.docx]

**Table 2.** Effects of metal ions (concentration 5 mM) and three chemical reagents on TtAbf62 ABF activity.

| **Chemicals** | **Relative activity (%)** | **Chemicals** | **Relative activity (%)** |
| --- | --- | --- | --- |
| **Control** | **100.00 ± 0.84** | **Ca^2+^** | **98.24 ± 0.08** |
| **Ni^2+^** | **54.18 ± 1.15** | **Fe^2+^** | **102.68 ± 3.22** |
| **Co^2+^** | **60.15 ± 1.15** | **Mg^2+^** | **68.97 ± 0.69** |
| **Al^3+^** | **51.65 ± 0.23** | **K^+^** | **92.18 ± 4.60** |
| **Na^+^** | **91.26 ± 1.76** | **Fe^3+^** | **101.61 ± 4.83** |
| **Mn^2+^** | **54.56 ± 1.53** | **SDS (0.1%)** | **ND** |
| **Zn^2+^** | **88.12 ± 1.53** | **EDTA (1mM)** | **89.27 ± 3.30** |
| **Cu^2+^** | **1.15 ± 0.84** | **Tween-20 (0.05%)** | **97.16 ± 1.23** |

Assays were performed in triplicate.

ND, not detected.
