# Supplementary material for: Highly efficient synergistic activity of an α-L-arabinofuranosidase for degradation of arabinoxylan in barley/wheat
Source: Front Microbiol. 2023 Nov 3;14:1230738. doi: 10.3389/fmicb.2023.1230738 (PMC10655120; doi:10.3389/fmicb.2023.1230738)
Supplement: Supplementary file 9 [file Table_3.docx]

**Table 3.** Yields of reducing sugars (mg/mL) obtained in 5 h during barley/wheat hydrolysis by TtAbf62 (α-L-arabinofuranosidase), Ttxy43 (β-xylosidase), and Taxy11 (endoxylanase) individually and in combinations as indicated.

| **Enzyme(s)** | **Reducing sugars yield**  **(mg/mL) on barley** | **Reducing sugars yield**  **(mg/mL) on wheat** |
| --- | --- | --- |
| **TtAbf62** | **0.00** | **0.14 ± 0.02** |
| **Ttxy43** | **0.00** | **0.02 ± 0.01** |
| **Taxy11** | **0.42 ± 0.01** | **0.65 ± 0.004** |
| **TtAbf62 + Ttxy43** | **0.00** | **0.08 ± 0.01** |
| **TtAbf62 + Taxy11** | **0.60 ± 0.05** | **0.80 ± 0.05** |
| **Taxy11 + Ttxy43** | **0.49 ± 0.03** | **0.74 ± 0.07** |
| **TtAbf62 + Taxy11 + Ttxy43** | **0.78 ± 0.05** | **1.08 ± 0.09** |
